# The dynamics of extracellular gadolinium-based contrast agent excretion into pleural and pericardial effusions quantified by T1 mapping cardiovascular magnetic resonance

**DOI:** 10.1186/s12968-019-0580-1

**Published:** 2019-11-14

**Authors:** Simon Thalén, Maren Maanja, Andreas Sigfridsson, Eva Maret, Peder Sörensson, Martin Ugander

**Affiliations:** 1 Department of Clinical Physiology, Karolinska Institutet and Karolinska University Hospital, Stockholm, Sweden; 2 Department of Cardiology, Karolinska University Hospital, Stockholm, Sweden; 3 University of Sydney, Northern Clinical School, Sydney Medical School, Sydney, Australia

**Keywords:** Cardiovascular magnetic resonance, Pericardial effusion, Pleural effusion, Gadolinium based contrast, T1 mapping, Vicarious excretion

## Abstract

**Introduction:**

Excretion of cardiovascular magnetic resonance (CMR) extracellular gadolinium-based contrast agents (GBCA) into pleural and pericardial effusions, sometimes referred to as vicarious excretion, has been described as a rare occurrence using T1-weighted imaging. However, the T1 mapping characteristics as well as presence, magnitude and dynamics of contrast excretion into these effusions is not known.

**Aims:**

To investigate and compare the differences in T1 mapping characteristics and extracellular GBCA excretion dynamics in pleural and pericardial effusions.

**Methods:**

Clinically referred patients with a pericardial and/or pleural effusion underwent CMR T1 mapping at 1.5 T before, and at 3 (early) and at 27 (late) minutes after administration of an extracellular GBCA (0.2 mmol/kg, gadoteric acid). Analyzed effusion characteristics were native T1, ΔR1 early and late after contrast injection, and the effusion-volume-independent early-to-late contrast concentration ratio ΔR1early/ΔR1late, where ΔR1 = 1/T1post-contrast - 1/T1native.

**Results:**

Native T1 was lower in pericardial effusions (*n* = 69) than in pleural effusions (*n* = 54) (median [interquartile range], 2912 [2567–3152] vs 3148 [2692–3494] ms, *p* = 0.005). Pericardial and pleural effusions did not differ with regards to ΔR1early (0.05 [0.03–0.10] vs 0.07 [0.03–0.12] s^− 1^, *p* = 0.38). Compared to pleural effusions, pericardial effusions had a higher ΔR1late (0.8 [0.6–1.2] vs 0.4 [0.2–0.6] s^− 1^, *p* < 0.001) and ΔR1early/ΔR1late (0.19 [0.08–0.30] vs 0.12 [0.04–0.19], *p* < 0.001).

**Conclusions:**

T1 mapping shows that extracellular GBCA is excreted into pericardial and pleural effusions. Consequently, the previously used term vicarious excretion is misleading. Compared to pleural effusions, pericardial effusions had both a lower native T1, consistent with lesser relative fluid content in relation to other components such as proteins, and more prominent early excretion dynamics, which could be related to inflammation. The clinical diagnostic utility of T1 mapping to determine quantitative contrast dynamics in pericardial and pleural effusions merits further investigation.

## Introduction

T1 mapping cardiovascular magnetic resonance imaging (CMR) before and after gadolinium based contrast agent (GBCA) administration has emerged as a useful tool for quantitative tissue characterization in the myocardium [1] but has unexplored potential to characterize the composition of serous fluids in the body. The composition of serous fluids such as pericardial and pleural effusions as well as ascites have been characterized non-invasively using x-ray computerized tomography (CT) [2–7] and CMR without T1 mapping [8, 9]. The pericardial space normally contains 15–50 ml of pericardial fluid [10]. Any larger amount is considered a pericardial effusion and may be due to different etiologies including malignancy, heart failure, infection or renal insufficiency [11]. Similarly, accumulation of fluid in pleural space is considered a pleural effusion, and pleural effusions can have similar etiologies as pericardial effusions [12].

Non-renal excretion of iodinated contrast agents (ICA) [13–17] as well as extracellular GBCA [18] has been referred to as vicarious excretion and is described as a rare occurrence. Vicarious excretion of ICA into both pleural and pericardial effusions have been reported using computed tomography (CT) [19–21] and one study noted that all patients that showed excretion of ICA into pericardial effusions followed a benign course [22]. Furthermore, excretion of extracellular GBCA into pleural effusions have also been reported using CMR and T1-weighted imaging approaches [23, 24].

ICA and extracellular GBCA have a similar extracellular distribution and renal clearance [25, 26]. A good agreement has been found comparing quantitative tissue characterization using cardiac CT and T1 mapping CMR [27] with ICA and extracellular GBCA, respectively. Whereas the presence of ICA directly increases the signal intensity in CT images, the presence of extracellular GBCA shortens T1 and yields an increase in signal intensity in T1-weighted images [28]. As the signal intensity in T1-weighted images is displayed in arbitrary units the changes in imaging characteristics can consequently only be determined semi-quantitatively. Recent advances in CMR imaging allow so called T1 mapping [29] whereby the T1 value of each pixel is determined and displayed as a T1 map [29]. The change in 1/T1 before and after contrast administration, called ΔR1, is linearly proportional to contrast agent concentration, and T1 mapping has proven highly sensitive to small changes in T1 [30]. Thus, T1 mapping provides the opportunity to perform a more sensitive and quantitative characterization of contrast dynamics in pericardial and pleural effusions.

The clinical implications and potential diagnostic utility of identifying contrast excretion into pericardial and pleural effusions by CMR remain largely unexplored. Furthermore, the presence, magnitude, and relative rate of extracellular GBCA excretion into pericardial and pleural effusions are not known. Therefore, the aim of the study was to investigate and compare the differences in T1 mapping characteristics and extracellular GBCA excretion dynamics in pleural and pericardial effusions.

## Methods

### Patient selection

**Fig. 1:**
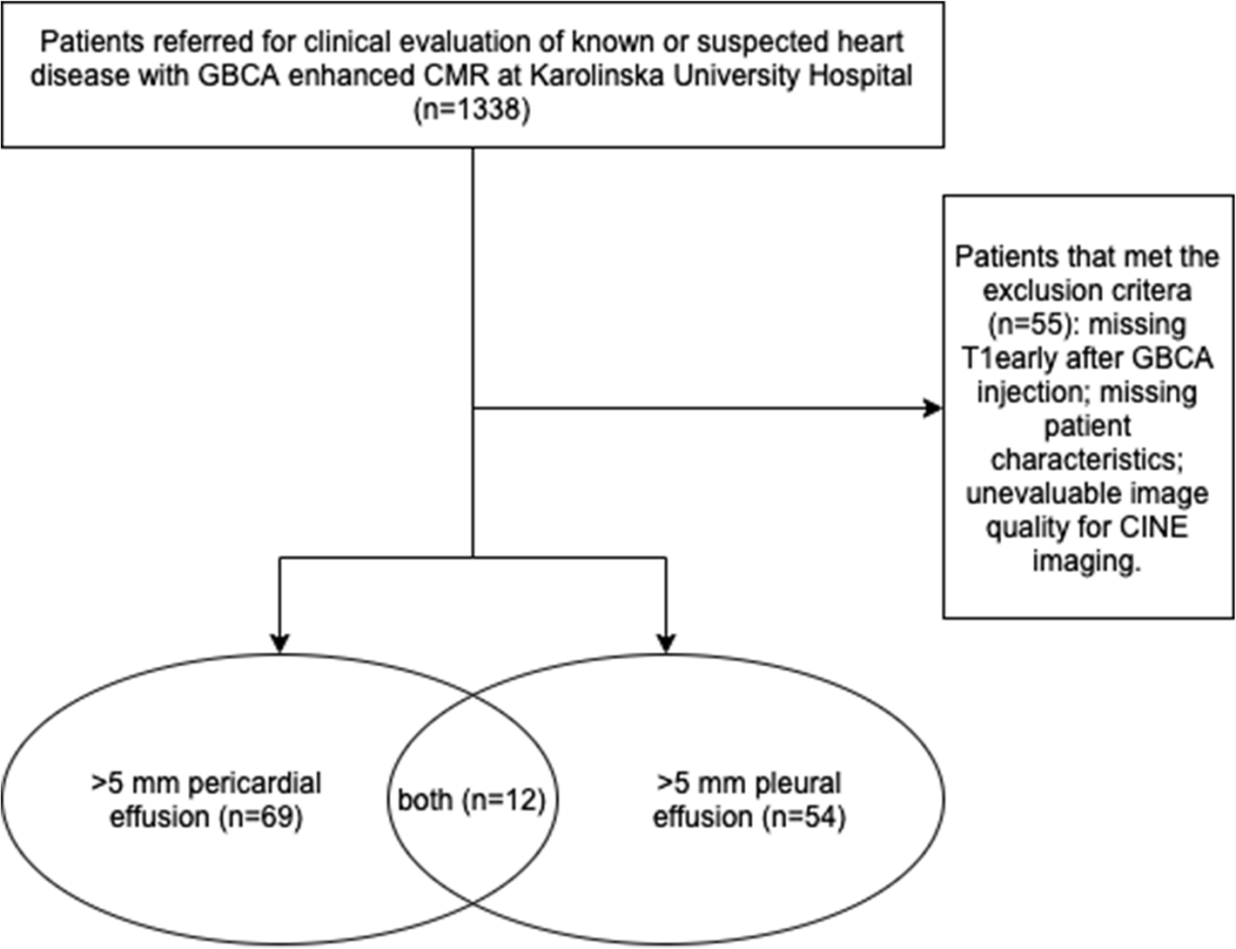
Flow chart of patient selection. GBCA, gadolinium based contrast agent

Consecutive patients referred for clinical evaluation of known or suspected heart disease with extracellular GBCA-enhanced CMR were retrospectively screened for inclusion. Inclusion criteria were at least 5 mm pericardial effusion and/or 5 mm pleural effusion in an end-diastolic cine 4-chamber image and T1 maps acquired in the same imaging slice before, early (approximately 3 min) and late (approximately 25 min) after administration of extracellular GBCA (0.2 mmol/kg, gadoteric acid, Dotarem®, Gothia Medical AB, Billdal, Sweden). The cutoff value for the size of a pericardial and pleural effusion was chosen with consideration to whether it was possible to reliably measure T1 values from the respective effusion, given the spatial resolution of the T1 maps. In the case of a bilateral pleural effusion, the right-sided effusion was used for analysis for reasons of reporting only one value per subject. The patient heart rate, presence of ascites and estimated glomerular filtration rate (eGFR) was noted. Exclusion criteria included missing early T1 map, missing patient characteristics and non-evaluable image quality. A flow chart describing the patient selection process is shown in Fig. 1.

### Image acquisition

T1 maps were acquired using a modified look-locker inversion recovery (MOLLI) sequence at 1.5 T (Aera, Siemens Healthineers, Erlangen, Germany). Images were acquired using a non-selective inversion pulse, balanced steady state free precession single shot read-out at end-diastole. Two inversions were made, five images acquired after the first inversion, followed by a three heart beats pause, and three images were acquired after the second inversion. Typical image acquisition parameters were: flip angle 35°, matrix size 256 × 136–158, slice thickness 8 mm, initial inversion time 129 ms, field of view 300-410 × 241–384 mm^2^, parallel imaging factor 2.

### Image analysis

**Fig. 2:**
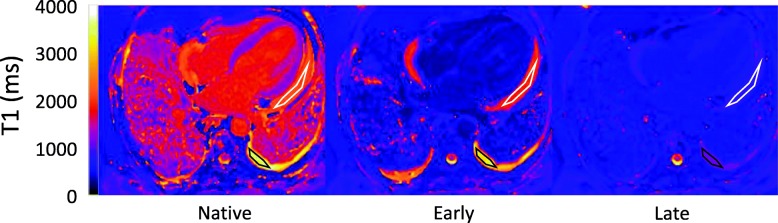
Example T1 maps taken before, early and late after GBCA administration. The white and black regions of interest illustrate how measurements were delineated in the pericardial and pleural effusions, respectively

Image analysis was performed based on manual delineation of regions of interest in effusions, myocardium and blood pool using a clinically used work station (IDS7, Sectra, Sweden). Each region of interest was delineated within the effusions, myocardium and blood pool while taking care to avoid partial volume effects in the edges of the region, as illustrated in Fig. 2. Analyzed variables in the respective effusions, blood pool and myocardium were native T1 (T1native), the difference between T1native and T1 acquired early (ΔT1early) and late (ΔT1late) after contrast injection, the change in R1 values (ΔR1early and ΔR1late) and the effusion-volume-independent early-to-late contrast concentration ratio (ΔR1early/ΔR1late), where ΔR1 = 1/T1post-contrast - 1/T1native. Extracellular volume fraction (ECV) was quantified at the late timepoint using the formula ECV = (1-hematocrit) (**Δ**R1 effusion) / (**Δ**R1 blood). The hematocrit was acquired by venous blood sampling on the day of CMR. An estimation of the GBCA concentration can be made using the formula C = ΔR1/r_1_, where r_1_ is the specific relaxivity of gadoteric acid which has previously been determined to be 3.6 L/mmol^− 1^/S^− 1^ at 1.5 T [31].

### Statistical analysis

Data are presented as median, interquartile range (IQR) and range, or mean and standard deviation as appropriate. Shapiro-Wilks test was used to assess normality. Differences were tested using Student’s t-test for normally distributed variables and the Mann-Whitney test for non-normally distributed variables. Correlations were evaluated using the Pearson correlation coefficient and simple linear regression was evaluated by the *R*^2^ values. Statistical comparisons were performed using SPSS (version 24, Statistical Package for the Social Sciences (SPSS), International Business Machines, Inc., Armonk, USA). A *p*-value of less than 0.05 was considered statistically significant.

## Results

Screening of 1338 consecutive patients undergoing CMR at a single university hospital between September 2013 and November 2015 identified a study population with a pericardial effusion, early and late post-contrast measurements of T1 in 69 (5%) patients (54 ± 16 years, 59% male). Similarly, screening identified a study population with a pleural effusion, early and late post-contrast measurements of T1 in 54 (4%) patients (59 ± 14 years, 59% male). Twelve patients had both a pericardial and a pleural effusion and 20 patients a bilateral pleural effusion. No subjects in the study population had ascites. The patient selection process is summarized in Fig. 1, and the characteristics of the study population are summarized in Tables 1 and 2.

**Table 1:** Baseline variables for the pericardial and pleural effusion groups

Characteristics	Pericardial effusion	Pleural effusion			*p*-value
Number, n	69	*	54	*	
Age, years	58	(46–65)	64	(52–71)	0.03
Male sex, n (%)	41	(59)	32	(59)	0.82
LVM, g	187	(153–240)	188	(143–236)	0.82
LVMI, g/m2	97	(83–120)	95	(83–120)	0.79
LVEDV, mL	156	(127–190)	150	(125–190)	0.72
LVEDVI, mL/m^2^	81	(69–88)	81	(65–100)	0.60
LVEF, %	47	(36–56)	44	(30–53)	0.22
BMI, kg/m^2^	25	(23–28)	25	(22–29)	0.52
Effusion size, mm	8	(5–9)	14	(6–10)	< 0.001

Continuous data are given as median (interquartile range) or number (%). *LVM* left ventricular mass, *LVMI* Left ventricular mass index, *BMI* Body mass index, *LVEDV* Left ventricular end-diastolic volume, *LVEDVI* Left ventricular end-diastolic volume index, *LVEF* Left ventricular ejection fraction, *LVM* Left ventricular mass, *LVMI* Left ventricular mass index. * *n* = 12 had both a pericardial and a pleural effusion

**Table 2:** Distribution of diagnoses by type of effusion

Diagnosis	Pericardial *n (%)*		Pleural *n (%)*	
Otherwise normal	3	(4)	2	(4)
IHD	21	(30)	18	(33)
Dilated LV	14	(20)	8	(15)
LVH	2	(3)	1	(2)
Dilated LV + LVH	2	(3)	1	(2)
Myocarditis	5	(7)	4	(7)
Tumor	2	(3)	0	
Reduced LVEF	3	(4)	3	(6)
Takotsubo	4	(6)	6	(11)
Pericarditis	3	(4)	3	(6)
Sarcoidosis	2	(3)	0	
Amyloidosis	2	(3)	3	(6)
Other	6	(9)	5	(9)
**Total**	69	(100)	54	(100)

*IHD* Ischemic heart disease, *dilated LV* Dilated left ventricle, *reduced LVEF* Reduced left ventricular ejection fraction, without ischemic heart disease or myocarditis, *LVH* Left ventricular hypertrophy; other, other diagnosis, such as non-compaction, vasculitis, postpartum cardiomyopathy, or indeterminate diagnosis

An example of T1 maps of a single patient, before, early and late after contrast administration is shown in Fig. 2.

**Fig. 3:**
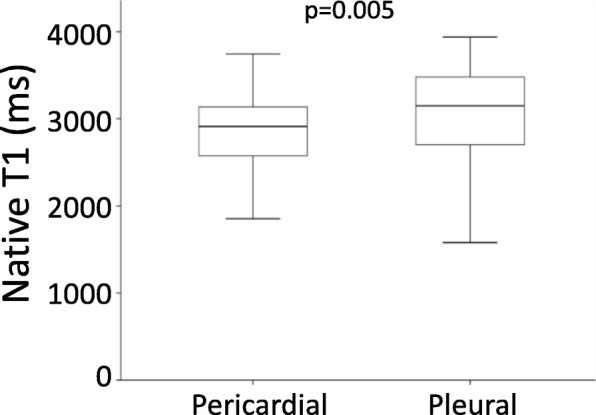
Boxplot of native T1 for pericardial and pleural effusions. The boxes indicate median and interquartile range, and whiskers the full range

Native T1 values were lower in pericardial effusions (2912 [2567–3152] ms, range 1855–3744, *n* = 69) compared to pleural effusions (3148 [2692–3494] ms, range 1481–3938, *n* = 54, *p* = 0.005), illustrating a lesser relative fluid content of pericardial effusions (Fig. 3). There was no relationship between heart rate and native T1 (*R*^*2*^ < 0.01, *p* = 0.35).

**Fig. 4:**
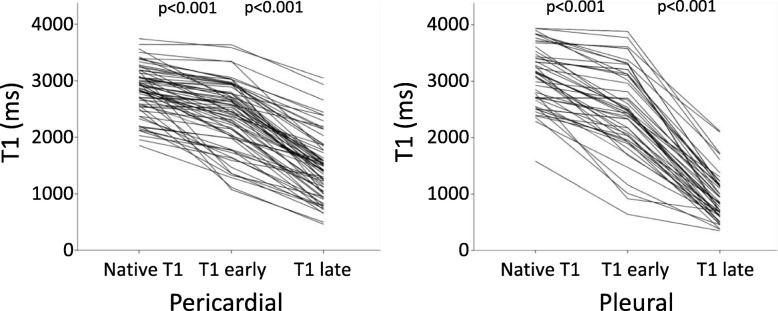
T1 measurements before, early and late after extracellular GBCA injection for each individual pericardial and pleural effusion subject, respectively. Note that all effusions had a reduction in T1 at least late after contrast injection, indicating that extracellular GBCA is excreted into all pericardial and pleural effusions

The changes in T1 values before and after GBCA administration for pericardial and pleural effusions are shown in Fig. 4. ΔT1late values of pericardial effusions were (1342 [1029–1681] ms, range 602–2504), illustrating that there was contrast excretion into all pericardial effusions, manifested as a substantial reduction in T1late after contrast administration (> 602 ms reduction in T1 for all). Similarly, ΔT1late values of pleural effusions were (2210 [1768–2508] ms, range 1234–3146), illustrating that there was contrast excretion into all pleural effusions, manifested as a substantial reduction in T1late after contrast administration (> 1234 ms reduction in T1 for all).

**Fig. 5:**
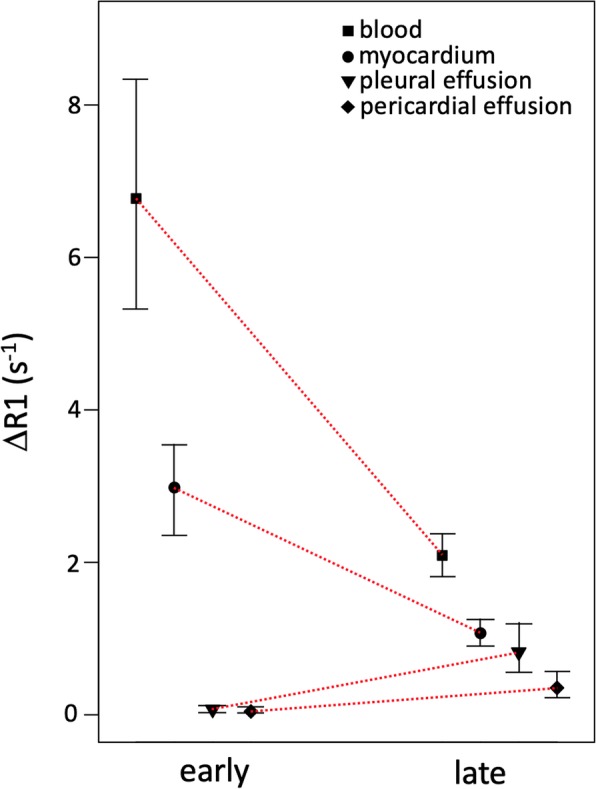
Plot of ΔR1 for the blood, myocardium, pleural effusion and pericardial effusion both early and late after contrast administration. Symbols denote the median, and whiskers the interquartile range. The red lines indicate change in ΔR1. ΔR1 is proportional to contrast agent concentration. Note how blood and myocardium have a relatively high contrast agent concentration early after contrast administration, which then decreases over time at the late time point. By comparison, both the pleural and pericardial effusions have measurable but low relative contrast agent concentrations early after contrast administration, which increase over time at the late time point, to a level that is lower than for blood and myocardium. These magnitudes and dynamics of relative contrast agent concentration suggest that the effusions do not establish a dynamic equilibrium with the blood. Thus, it would be misleading to calculate the extracellular volume fraction for the effusions

There was no difference in ΔR1early values between pericardial effusions (0.045 [0.026–0.100] s^− 1^, range 0.0003–0.5932) and pleural effusions (0.073 [0.028–0.121] s^− 1^, range 0.0008–0.9300, *p* = 0.38). ΔR1late was lower in pericardial effusions (0.35 [0.23–0.59] s^− 1^) compared to pleural effusions (0.82 [0.56–1.20] s^− 1^, *p* < 0.001). ΔR1early and ΔR1late are displayed in Fig. 5.

**Fig. 6:**
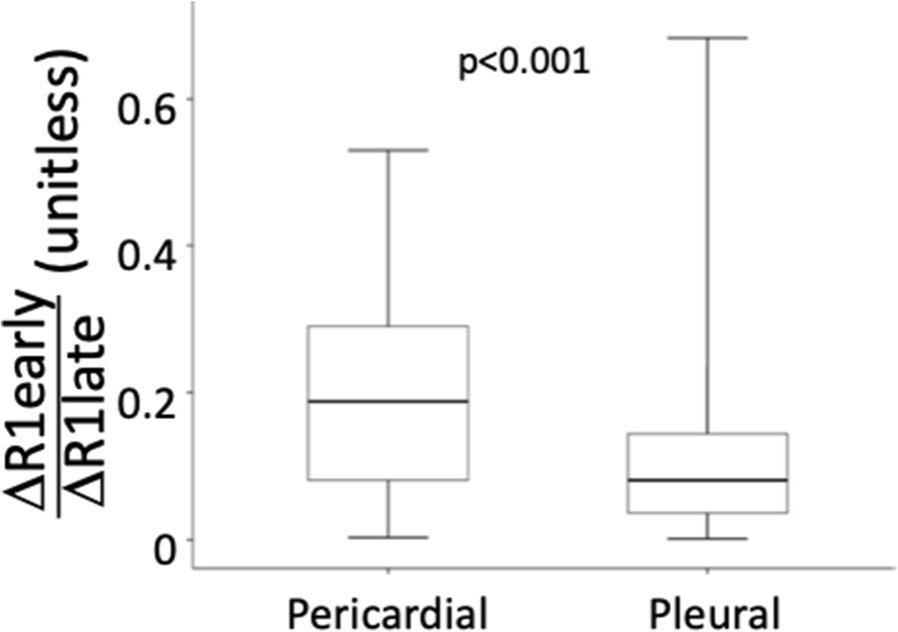
Boxplot of the ratio formed by ΔR1early and ΔR1late for pericardial and pleural effusions. This ratio is independent of the volume of the effusion. The boxes indicate median and interquartile range, and whiskers –the full range

The early-to-late contrast concentration ratio (ΔR1early/ΔR1late) was higher in pericardial effusions (0.180 [0.080–0.290], range 0.003–0.530), compared to pleural effusions (0.080 [0.003–0.144], range 0.002–0.683, *p* < 0.001), indicating that pericardial effusions have more prominent early excretion dynamics. The early-to-late contrast concentration ratio is displayed in Fig. 6.

**Fig. 7:**
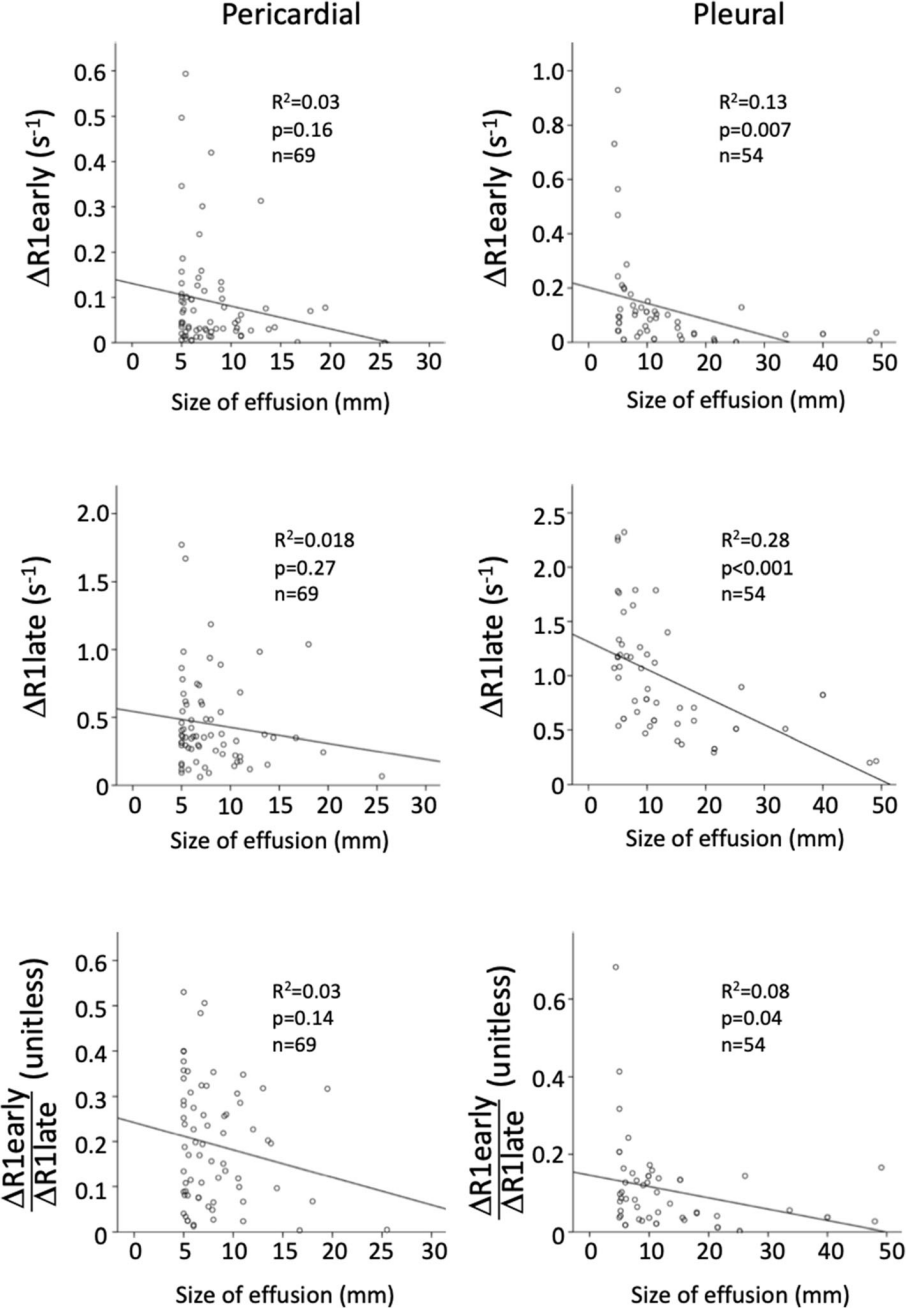
Linear correlations between the size of effusion (mm) and ΔR1early (s^−1^), ΔR1late (s^−1^), unitless volume-independent ratio ΔR1early/ΔR1late for the pericardial (left) and pleural effusion groups (right)

There was a weak correlation between the size of pleural effusion and ΔR1early (*R*^*2*^ = 0.13, *p* = 0.007), ΔR1late (*R*^*2*^ = 0.28, *p* < 0.001), and the early-to-late contrast concentration ratio ΔR1early/ΔR1late (*R*^*2*^ = 0.08, *p* = 0.04). This indicates that larger pleural effusions might have a lower concentration of extracellular GBCA, as measured both early and late after injection. Also, larger pleural effusions had more prominent late excretion. Pericardial effusion size did not correlate with quantitative measures. The effusion size plotted against ΔR1early, ΔR1late and the early-to-late contrast concentration ratio are displayed in Fig. 7.

In the bilateral pleural effusions (*n* = 20), the left- and right-sided effusions did not differ with regards to in any of our analyzed variables (*p* > 0.05 for native T1, ΔR1 early, ΔR1 later, and ΔR1early/ΔR1 late).

The ΔR1 of the blood pool early after contrast agent administration was (6.8 [5.3–8.3] s^− 1^, range 1.6–15.0), and decreased at the late time point (2.1 [1.8–2.4] s^− 1^, range 1.2–3.5, *p* < 0.001). The ΔR1 of the myocardium early after contrast agent administration was (3.0 [2.4–3.5] s^− 1^, range 0.1–7.7), and also decreased at the late time point (1.1 [0.9–1.2] s^− 1^, range 0.6–2.2, *p* < 0.001). ΔR1 is directly proportional to relative contrast concentration, and consequently the blood pool and the myocardium had a relatively high contrast agent concentration early after administration, which decreased over time between the early and late time points. By comparison, both types of effusions had an increasing relative contrast agent concentration over time.

ECV values for effusions do not necessarily represent the extracellular space of the respective effusion, but they are reported herein for pragmatic purposes to reflect the magnitude and range with which the extracellular GBCA distributes into these effusions at the late timepoint. ECV at the late timepoint for the pericardial effusions was 9 [6–14] %, range 2–61%, and the ECV for the pleural effusions was 24 [14–36] %, range 6–59%.

Estimated GBCA concentration at the early timepoint was (12.4 [7.1–28.7] mmol/mL, range 0.1–164.8) for the pericardial effusions and (20.0 [7.9–33.8] mmol/mL, range 0.2–258.3) for the pleural effusions. Estimated GBCA concentration at the late timepoint was (98.2 [63.3–154.7] mmol/mL, range 16.9–491.7) for the pericardial effusions and (228.5 [154.8–331.5] mmol/mL, range 55.9–645.1) for the pleural effusions.

There was no relationship between renal function measured as eGFR in ml/min/1.73m^2^ and ΔR1early (*R*^*2*^ = 0.01, *p* = 0.82) or ΔR1late (*R*^*2*^ = 0.01, *p* = 0.89) of the pericardial effusions. Similarly, there was no relationship between renal function and ΔR1early (*R*^*2*^ = 0.02, *p* = 0.87) or ΔR1late (*R*^*2*^ = 0.02, *p* = 0.96) of the pleural effusions.

## Discussion

The present study is the first to quantify the T1 of pericardial and pleural effusions before and after intravenous extracellular GBCA administration*.* T1 mapping shows that all patients with pericardial effusion and all patients with pleural effusion clearly exhibit extracellular GBCA excretion into the effusion fluid. For both pericardial and pleural effusions, there is a considerable variability in native T1, suggesting a variable protein content, and the early/late contrast concentration ratio, suggesting a variable rate of excretion, likely due to the variable etiologies of the effusions. Also, larger pleural effusions were associated with a decreasing early-to-late contrast concentration ratio indicating more prominent late, rather than early, excretion.

T1 mapping revealed that, compared to pleural effusions, pericardial effusions have a lower native T1. These differences in native T1 values between pleural and pericardial effusions highlight their different biochemical composition. The proteins present in serous fluids have paramagnetic properties, although not as potent as those of extracellular GBCA. Nonetheless, any proteins present will shorten the T1 of nearby protons. Our results suggest that the protein content of pericardial effusions is greater than that of pleural effusions. This is in line with previous studies, as a majority of pericardial effusions were found to be exudates due to high protein content [32, 33] when evaluated using the Light criteria [34].

Pericardial and pleural effusions did not differ in ΔR1early, but pleural effusions had higher ΔR1late, resulting in a lower ΔR1early/ΔR1late that indicates more prominent late excretion dynamics in comparison to early. It is important to note that ΔR1early and ΔR1late are both dependent on effusion volume, whereas ΔR1early/ΔR1late is independent.

The first diagnostic step in determining the cause of a pleural effusion is often to determine if the effusion is an exudate or a transudate following thoracocentesis and biochemical analysis [12].

By analogy, the same differentiation holds for pericardial effusions and pericardiocentesis, but the biochemical analysis is less documented and some argue against [32, 33], and some for [35], its diagnostic usefulness.

One study found that the amount of extracellular GBCA excretion into pleural effusions, as measured by T1-weighted imaging, had a sensitivity of 83% and specificity of 100% for determining whether an effusion was a transudate or an exudate [24]. Transudates did not differ in T1-weighted signal intensities after extracellular GBCA injection, and the authors presumed that the increased pleural permeability of exudates contributed to excretion of extracellular GBCA into the effusion [24]. Our results are based upon more sensitive quantitative imaging, showing that all pleural effusions exhibit contrast excretion. It is unlikely that all pleural effusions measured in our study were exudates, and thus the use of contrast excretion into an effusion to differentiate between transudate and exudate may be more nuanced than initially thought.

Vicarious excretion was first used to describe a phenomenon whereby urographic contrast agents were to some extent excreted by the biliary pathway, and observed as attenuation of the gallbladder on x-ray film or by CT [13–17]. By contrast, our results show that extracellular GBCA excretion into pericardial and pleural effusions always occurs, and thus is physiological rather than vicarious. Our data support the conclusion that the term vicarious excretion should be abandoned with regards to describing extracellular GBCA excretion into pericardial and pleural effusions.

Studies using CT have found higher attenuation in exudates than in transudates in pleural effusions with varying degrees of overlap [2, 3, 7]. Another study found the opposite, a higher attenuation in transudates than in exudates [5]. Also, in these studies, ICA was only occasionally administered [2], administered but not analyzed [7], administered but no difference in pre- and postcontrast attenuation was found [3] or administered and a difference was found between pre- and postcontrast attenuation, but only for transudates [5]. For pericardial effusions, one study using CT found a difference in pre- and postcontrast attenuation in some effusions and noted that they all followed a benign course [22], whereas another found no difference [4]. In summary, the results with regards to contrast dynamics at CT have shown considerable variability.

The results from CT imaging described above are in contrast to our results, showing extracellular GBCA excretion into all pericardial and pleural effusions in our study. Although CT and T1 mapping CMR are different methods of measurement, ICA and extracellular GBCA are similar in their distribution and renal clearance [25, 26], and a good agreement has been found between the two in the setting of quantitative tissue characterization [27].

The amount of extracellular GBCA excreted into the pericardial or pleural space will likely depend on multiple factors including pericardial or pleural permeability, the volume of effusion, amount of extracellular GBCA injected, renal function, and hydrostatic as well as oncotic pressures. One could hypothesize that as the pericardial or pleural permeability increases, due to for instance inflammation, excretion of extracellular GBCA would be both greater and faster.

T1 measurements are routinely used to calculate the myocardial ECV, which has been proven a useful marker of various myocardial pathologies [36]. The current study found that the relative contrast agent concentration of the blood pool and the myocardium both decrease over time. With the exception of myocardium with acute microvascular obstruction, there is a rapid exchange of contrast between the myocardium and blood, leading to a dynamic equilibrium and concomitant decreases in relative contrast agent concentration over time after an intravenous bolus due to renal clearance [37]. However, we found that both effusion types increased in relative contrast concentration over time after intravenous bolus contrast administration. While the blood has an extracellular space equaling 1-hematocrit, serous effusions typically have very few cells, and would be expected to have a contrast agent concentration roughly double that of blood. However, the relative contrast agent concentration in the effusions did not exceed that of the blood at the late time point. Taken together, these data provide evidence that a dynamic equilibrium is not achieved between the contrast agent concentration in the blood and either of the effusion compartments. Thus, reporting relative contrast agent distribution calculated as ECV for these compartments is misleading and should be avoided. For reasons of completeness we have reported the ECV values at the late timepoint for the pericardial and pleural effusions, respectively. Notably, all effusions had ECV values greater than zero, reflecting the finding that the extracellular GBCA was measurably distributed into all effusions. However, the values had considerable variability, likely relating to the varying nature, physiology, and dynamics of contrast distribution into the effusions.

### Limitations

The retrospective nature of the study meant that there was no access to biochemical analyses of the fluid and the etiology of the effusions could not be definitively determined. It is therefore not possible to discern whether an increased or decreased extracellular GBCA excretion was associated with being of exudative or transudative etiology. Furthermore, we did not have access to outcomes data. The results of the current study illustrate the large variation in quantitative imaging characteristics of pericardial and pleural effusions, which presumably at least in part can be related to etiology. These results provide justification for future prospective studies that study the etiology and biochemical composition of the effusions, as well as clinical outcomes.

A further limitation of the current study is that all patients were injected with an amount of extracellular GBCA in proportion to their body weight, but the quantitative imaging characteristics did not control for serum oncotic or hydrostatic pressure.

We only measured the size of the effusion in the four-chamber view as our study protocol did not include other views acquired at all time points before, early, and late after contrast administration. Visualizing and measuring T1 of effusions may be more comprehensive when using additional imaging views.

The inclusion criteria of 5 mm of effusion was arbitrarily chosen with regards to whether it was possible to reliably measure T1 values from the respective effusion. As this study is the first to measure T1 values in pericardial and pleural effusions using T1 mapping, the exact number of mm that are required to reliably measure the T1 values of pericardial or pleural effusions is an open question. We chose 5 mm for purposes of consistency based on clinical judgement.

The extracellular GBCA used in our study was gadoteric acid and we did not investigate or compare any other GBCA. As the biodistribution of all extracellular GBCA are considered equivalent, we would not expect different results with regards to contrast distribution dynamics and relative contrast agent concentrations using other types of extracellular GBCA [38].

This study has a selection bias as the patients referred to undergo a CMR study are much more likely to suffer from a heart condition than a respiratory condition. For example, it is much less likely that a patient suffering from a pleural effusion due to pneumonia would be imaged by CMR at our center, compared to a patient suffering from pleural or pericardial effusion due to heart failure or inflammatory heart disease. As such, our study population is biased towards cardiac-related pathologies. The study population is also biased towards smaller pericardial effusions since larger effusions are more likely to be evaluated emergently using echocardiography.

A limitation of the current study is that the late T1 measurements were acquired approximately 30 min after contrast administration. The quantitative contrast dynamics in effusions have not been comprehensively explored, and are largely unknown. T1 measurements from multiple later timepoints after contrast administration may be necessary to fully understand the physiology of serous compartment dynamics, and future studies to address these open questions are justified.

A longer T1 means that a longer time is needed for the tissue or fluid to achieve complete longitudinal relaxation. In the MOLLI acquisition scheme, which utilizes multiple inversions, incomplete relaxation between the inversions may have a negative effect the on accuracy and precision of the estimated T1 value. In this study, the time between inversions was defined in terms of a number of heartbeats, meaning that higher heart rates shortened the time available to achieve complete longitudinal relaxation. We did not find any relationship between heart rate and native T1 in our study population, indicating that high heart rates did not contribute to any systematic bias compared to low heart rates. This finding is further corroborated by our phantom data, suggesting that high heart rates resulted in a loss of precision, but no systematic bias (see Appendix).

## Conclusion

T1 mapping showed that intravenous extracellular GBCA is excreted into both pericardial and pleural effusions in all patients. Consequently, the previously used term vicarious excretion is misleading. Compared to pleural effusions, pericardial effusions had both a lower native T1, consistent with lower relative fluid content in relation to other components such as proteins and more prominent early excretion dynamics. The clinical diagnostic consequences of T1 mapping and quantitative contrast dynamics in pericardial and pleural effusion merit further prospective investigation.

## Data Availability

Please contact author for data requests.

## Appendix

### Phantom study

The MOLLI acquisition scheme used in this study is known to have some heart rate dependency [30]. Thus, we investigated the effect of heart rate on T1 measurements of pericardial and pleural fluids using a phantom. A plastic bottle containing 500 ml of sterile water was used to emulate the long T1 values of a pericardial or pleural effusion. To obtain a reference T1 value, free from heart rate dependence, the phantom was first imaged using a B1 corrected variable flip angle T1 mapping sequence. The phantom was then imaged several times using the modified Look-Locker inversion recovery (MOLLI) sequence previously described. Electrocardiogram signals were simulated with RR-intervals ranging from 400 to 1500 ms in 100 ms increments. Measurements were obtained using a circular region-of-interest 4 cm in diameter placed in the center of the bottle. The percentage of relaxation r immediately before the second inversion of the MOLLI sequence using the following formula:

$$ \mathrm{r}=1-{\mathrm{e}}^{-\frac{\mathrm{t}}{{\mathrm{T}}_1^{\ast }}} $$

Where t was the time between the first and second inversion in milliseconds, i.e. 8 times the RR-interval for a MOLLI readout, and T1* the apparent relaxation time constant in milliseconds as measured by the MOLLI sequence.

The acquisition parameters of the B1 corrected variable flip angle sequence used in the phantom study was: flip angle 3° and 15°, matrix size 156 × 256 field of view 380 mm * 309 mm.

The reference T1 value of the sterile water phantom was measured to 3011 ms. The results of variable RR-intervals on T1 measurements in the phantom using MOLLI are reported in Table 3. For RR-intervals below 600 ms, the MOLLI sequence failed to report T1 values, presumably due to lack of convergence of the curve fitting algorithm.

**Table 3:** The relationship between heart rate and T1 mapping measurements

RR (ms)	HR (bpm)	T1 (ms)	Curve fit uncertainty in SD (ms)	T1* (ms)	Relaxation (%)
600	100	3363	1000	1000	98%
700	86	2948	882	1530	96%
800	75	2853	464	1949	94%
900	67	2808	256	2198	95%
1000	60	2818	158	2389	95%
1100	55	2827	106	2498	96%
1200	50	2859	73	2604	97%
1300	46	2867	54	2612	97%
1400	43	2878	41	2645	98%
1500	40	2910	33	2674	98%

The T1 values of a phantom with a reference T1 value of 3011 ms imaged using MOLLI acquisitions with increasing heart rates (HR). The curve fit uncertainty was obtained from the SD maps provided by the MOLLI reconstruction, T1* is the apparent T1 relaxation time constant and relaxation the percentage of relaxation after 8 RR-intervals, immediately before the second inversion of the MOLLI sequence

## Abbreviations

CMR: Cardiovascular magnetic resonance
CT: Computed tomography
ECV: Extracellular volume fraction
eGFR: Estimated glomerular filtration rate
GBCA: Gadolinium-based contrast agents
ICA: Iodinated contrast agent
IQR: Interquartile range
MOLLI: Modified look-locker inversion recovery
